# Ferroptosis regulation by SGLT2 inhibitors: mechanisms and clinical benefits in diabetic kidney disease

**DOI:** 10.3389/fphar.2026.1808167

**Published:** 2026-04-07

**Authors:** Shumin He, Zongheng Wu, Sumei Li

**Affiliations:** 1 The Graduate School of Fujian Medical University, Fuzhou, Fujian, China; 2 Department of Endocrinology, The First Hospital of Putian City, Putian, Fujian, China; 3 Medical Department, Putian University, Putian, Fujian, China

**Keywords:** diabetic kidney disease, ferroptosis, kidney biomarkers, pharmacological intervention, SGLT2 inhibitors

## Abstract

Diabetic kidney disease (DKD) is a leading cause of end-stage renal disease and remains inadequately managed by conventional therapies focused on glucose control and hemodynamics. Growing evidence suggests that ferroptosis, an iron-dependent form of regulated cell death driven by lipid peroxidation, plays a critical role in renal tubular injury, oxidative stress, and fibrosis in DKD. The diabetic renal microenvironment is characterized by dysregulated iron handling, impaired antioxidant capacity, and lipid metabolic reprogramming, all of which converge to promote ferroptotic vulnerability. Sodium–glucose cotransporter 2 (SGLT2) inhibitors have emerged as cornerstone agents in DKD treatment, conferring substantial renoprotective benefits beyond glycemic control. Recent studies indicate that SGLT2 inhibitors regulate ferroptosis through different mechanisms. The effects include correcting iron imbalances associated with hypoxia, enhancing fatty acid β-oxidation, and strengthening the antioxidant defense system by activating the AMP-activated protein kinase (AMPK)-nuclear factor erythroid 2–related factor 2 (*NRF2*)-glutathione peroxidase 4 (*GPX4*) axis. Furthermore, ferroptosis-related biomarkers in serum and urine display disease stage–dependent alterations, highlighting their potential utility for patient stratification and therapeutic response prediction. This review elucidates how SGLT2 inhibitors promote renal protection in DKD by regulating ferroptosis, supported by both mechanistic insights and clinical evidence. It also addresses unresolved issues in its current clinical application. Furthermore, this work proposes a promising pharmacological strategy for the prevention and treatment of DKD.

## 1 Introduction

Diabetes has become one of the major public health burdens in the world. According to the Diabetes Atlas released by the International Diabetes Federation (IDF), the prevalence of diabetes among adults aged 20 to 79 around the world has reached 11.1%, and about 589 million people have been affected (Duncan et al., 2025). Diabetic kidney disease (DKD) is one of the most common and disabling chronic microvascular complications of diabetes and has become the leading cause of end-stage kidney disease (ESKD) (de Boer et al., 2022). In addition, chronic kidney disease (CKD) substantially increases the risk of cardiovascular events and all-cause death. Data from the Global Burden of Disease (GBD) study indicate that kidney failure contributes to approximately 11.5% of global cardiovascular mortality (Collaborators, 2025). The concept of “cardio-renal metabolic syndrome” proposed by the American Heart Association (AHA) further highlights the bidirectional pathological interactions and shared adverse outcomes among metabolic disorders, CKD, and cardiovascular disease (Young and Eknoyan, 2024).

Although intensified glycemic control, blood pressure management, and inhibition of the renin–angiotensin system (RAS) can slow DKD progression, its clinical course remains highly heterogeneous. A substantial proportion of patients continue to progress to advanced CKD or ESKD despite standard therapy (De Boer et al., 2022). This phenomenon shows that traditional intervention strategies focusing on metabolism and hemodynamics cannot completely prevent the continued deterioration of DKD. Therefore, it is of urgent significance to clarify the core pathological mechanism of the early stage of DKD, especially the molecular link between renal tubular injury and fibrosis.

In the field of procedural cell death, ferroptosis is a regulated form of cell death that depends on iron ions and lipid peroxidation, which is considered to be related to the development of a variety of diseases, including neurodegenerative diseases, cancer, acute kidney injury (AKI) and chronic kidney disease (Jiang et al., 2021). Renal tubular epithelial cells are particularly susceptible to ferroptosis due to their high demand for aerobic metabolism and active lipid metabolism (Li et al., 2023). Both experimental and clinical studies have reported iron accumulation, enhanced lipid peroxidation, and reduced glutathione peroxidase 4 (*GPX4*) expression in DKD kidney tissue, supporting ferroptosis as a potential mechanistic link between tubular injury, oxidative stress, and renal fibrosis (Wu and Chen, 2022).

From the perspective of intervention, identifying ferroptosis regulators in clinically available drugs is considered a strategy with great potential for transformation. Recent studies show that sodium-glucose cotransporter 2 (SGLT2) inhibitors can not only protect the kidneys by reducing blood sugar, improving hemodynamics and reducing proteinuria (Martinez Leon et al., 2026), but also inhibit ferroptosis by restoring iron homeostasis, limiting lipid peroxidation and enhancing antioxidant systems (Tsai et al., 2025). These effects appear to be at least partly independent of glycemic control in DKD models. This mechanistic insight provides a new biological framework for understanding the cardiorenal benefits of SGLT2 inhibitors and supports the development of ferroptosis-oriented therapeutic strategies for DKD. Therefore, this review aims to systematically summarize the pathological mechanisms of ferroptosis and its evidence supporting ferroptosis involvement in DKD, to further explore the hierarchical regulatory effects of SGLT2 inhibitors on ferroptosis-related pathways, and to discuss the current evidence and potential for clinical translation.

## 2 Ferroptosis in DKD: pathogenesis and evidence

### 2.1 Molecular and biochemical features

First proposed by Stockwell and colleagues in 2012,ferroptosis is a regulated form of cell death driven by iron dependency and lipid peroxidation (Dixon et al., 2012). Its morphological features differ greatly from those of classical cell death types, such as apoptosis and necrosis. It is characterized by mitochondrial contraction, increased mitochondrial membrane density, and a reduction or loss of mitochondrial cristae. There are no typical cell apoptotic characteristics, including chromatin condensation and plasma membrane foaming (Chen et al., 2024).

At the biochemical level, ferroptosis is driven by three core processes. Disruption of iron homeostasis leads to the accumulation of labile iron (Fe^2+^) in cells. Excess Fe^2+^ catalyzes the production of hydroxyl radicals (·OH) through the Fenton reaction, thus triggering a chain reaction of lipid peroxidation (Dixon et al., 2012; Dixon and Olzmann, 2024). In parallel, increased activity of lipid metabolic enzymes, including acyl-CoA synthetase long-chain family member 4 (*ACSL4*) and lysophosphatidylcholine acyltransferase 3 (*LPCAT3*), promotes the enrichment and remodeling of polyunsaturated fatty acids (PUFAs) in membrane phospholipids (Yang and Stockwell, 2016; Lee et al., 2021). This process increases the availability of oxidation sensitive lipid substrates. In addition, impairment of *GPX4*–glutathione (GSH) antioxidant system will damage the detoxification of lipid hydroperoxides, resulting in the fatal accumulation of lipid peroxidation products (Dodson et al., 2019).

### 2.2 Pathological mechanisms in DKD

**FIGURE 1:**
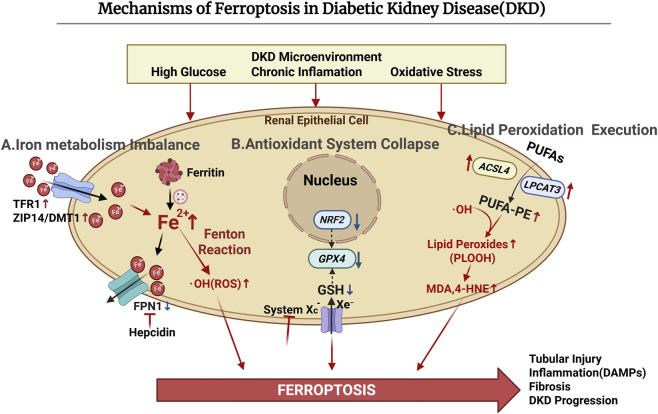
Molecular mechanisms underlying ferroptosis activation in renal tubular epithelial cells within the diabetic kidney disease microenvironment.

In DKD, the susceptibility to ferroptosis is jointly driven by iron metabolism disorder, impaired antioxidant defenses, and enhanced lipid peroxidation (Figure 1).

#### 2.2.1 Dysregulation of renal iron homeostasis

DKD is accompanied by serious disorders of iron metabolism and redox balance, making renal cells highly susceptible to ferroptosis. Hyperglycemia, inflammation, and metabolic disorders collectively disrupt the balance among iron uptake, storage, and export. As a result, labile iron accumulates in renal cells, providing a catalytic basis for lipid peroxidation (Wang W. et al., 2025).

In terms of iron uptake, diabetic conditions increase the expression of ZRT/IRT-like protein 14 (ZIP14), divalent metal transporter 1 (DMT1), and transferrin receptor 1 (TFR1). This increase makes more iron enter the kidney cells and causes iron buildup (Zhang et al., 2007; Wu et al., 2022; Kumar et al., 2024). Also, iron cannot get out easily. A high glucose environment breaks down ferroportin 1 (FPN1) and stops iron from leaving the cells (Zhao et al., 2024). Inflammatory cytokines such as interleukin-6 (IL-6) further induce hepcidin synthesis, which suppresses FPN1-mediated iron export and results in the continuous accumulation of renal iron (Wrighting and Andrews, 2006; Moulouel et al., 2013; Thévenod and Wolff, 2016; Zhao et al., 2024). In addition, iron storage is also affected. High blood sugar and oxidative stress trigger ferritinophagy, a process controlled by nuclear receptor coactivator 4 (*NCOA4*). This process releases stored iron into the labile iron pool and further increases Fe^2+^ levels in cell (Hou et al., 2016). Excess Fe^2+^ subsequently promotes lipid peroxidation through the Fenton reaction, thereby driving the execution phase of ferroptosis (Zhao, 2023).

#### 2.2.2 Impairment of antioxidant defense systems

The development of ferroptosis in DKD is closely related to the failure of antioxidant defense systems. This impairment mainly involves the System Xc^−^/GSH/*GPX4* axis and nuclear factor erythroid 2-related factor 2 (*NRF2)*-dependent transcriptional network (Wu and Chen, 2022).

System Xc^−^ (*SLC7A11*/*SLC3A2*) takes up cysteine to synthesize GSH(Li et al., 2022). GSH is a vital cofactor for *GPX4* to reduce phospholipid hydroperoxides (Friedmann Angeli and Conrad, 2018; Liu et al., 2022). In DKD, lower System Xc^−^ activity leads to GSH consumption (Li et al., 2022). This stops the clearance of lipid peroxides and drives ferroptosis (Li et al., 2022; Liu et al., 2022; Seibt et al., 2024). *NRF2* plays a central role in maintaining cellular redox balance and limiting ferroptosis. It starts the expression of several antioxidant genes, including *SLC7A11*, *GPX4*, and heme oxygenase 1(*HMOX1*) (Dodson et al., 2019). However, in DKD, endoplasmic reticulum stress speeds up the destruction of *NRF2* through the XBP1–Hrd1 axis. This impairs antioxidant defenses and boosts lipid peroxidation (Liu et al., 2023). Studies show that inhibitors like ferrostatin-1 reduce this damage, proving that ferroptosis signals are pathological in DKD (Kim et al., 2021).

Beyond the System Xc^−^/GSH/*GPX4* axis, other pathways also detoxify lipid radicals. For example, the ferroptosis suppressor protein 1 (FSP1) system works on the cell membrane (Doll et al., 2019). Also, the dihydroorotate dehydrogenase (DHODH) system works on the mitochondrial membrane (Mao et al., 2021). They both help get rid of these radicals. When oxidative stress is too heavy or *GPX4* is broken, these systems can only give partial safety (Bersuker et al., 2019; Mao et al., 2021). They usually fail to stop the damage completely.

#### 2.2.3 Lipid peroxidation and subcellular damage

Lipid peroxidation is the main cause of ferroptosis. In DKD, three main factors control this: changes in lipid substrates, iron-based oxidation, and weak antioxidant defenses (Yang and Stockwell, 2016).

At the substrate level, phosphatidylethanolamines containing polyunsaturated fatty acids (PUFA-PEs), such as arachidonic acid–PE (AA-PE) and adrenic acid–PE (AdA-PE), are the main oxidation targets (Zou et al., 2020). Lipidomic studies in DKD have shown marked remodeling of the lipid composition in renal tubular cells (Dixon et al., 2015). *ACSL4* and *LPCAT3* are the enzymes responsible for this (Dixon et al., 2015; Reed et al., 2022). They move PUFAs into membrane phospholipids, setting the stage for oxidation (Dixon et al., 2015). Next, accumulated Fe^2+^ helps produce phospholipid radicals and hydroperoxides (PLOOH) (Conrad and Pratt, 2019; Zhao, 2019). This causes membrane injury and signal errors. When *GPX4* activity is reduced, PLOOH breaks down into reactive aldehydes, such as malondialdehyde (MDA) and 4-hydroxynonenal (4-HNE) (Ayala et al., 2014). These molecules harm proteins and nucleic acids, triggering fibrosis and worsening renal injury (Ayala et al., 2014; Kim et al., 2021).

Lipid peroxidation also shows subcellular amplification effects. In the endoplasmic reticulum, PLOOH starts the PERK–eIF2α–ATF4–CHOP pathway and suppresses *NRF2* signals, further ruining the redox balance (Dixon et al., 2014). In mitochondria, lipid oxidation causes loss of membrane potential, increases reactive oxygen species (ROS) and promotes epithelial–mesenchymal transition (EMT) (Wang et al., 2016; Liu et al., 2023). This process is tied to renal interstitial fibrosis (Lyu et al., 2025).

### 2.3 Experimental and clinical evidence

The current research evidence covers many levels such as cellular models, animal studies, and clinical samples, suggesting that ferroptosis plays an important pathogenic role in kidney injury associated with DKD.

At the cellular level, high-glucose stimulation of HK-2 cells induces iron to build up, increases lipid oxidation and lowers *GPX4*. O-GlcNAcylation further stabilizes *ACSL4,* thereby promoting ferroptosis, indicating that hyperglycemia can directly activate this cell death pathway (Qin et al., 2025). Metabolic byproducts such as 2-deoxy-D-ribose also trigger ferroptosis by degrading the core subunit of System Xc^−^ (xCT) and consuming GSH (Kim et al., 2023). Single-cell transcriptomic and multi-omics analyses have shown that ferroptosis-related gene characteristics are enriched in specific proximal renal tubular subgroups during early type 2 DKD (Tsai et al., 2023; Dong et al., 2024). These changes display a causal association with disease progression.

In animal models, including db/db mice, streptozotocin (STZ)-induced diabetic models, and non-obese diabetic (NOD) mice, renal tissues show increased iron content, elevated levels of MDA and *ACSL4*, and reduced *GPX4* expression, which are the signature features of ferroptosis (Wang et al., 2020; Stancic et al., 2025). Genetic studies provide even deeper insights. They show that a lack of protein arginine methyltransferase 6 (PRMT6) drives lipid peroxidation by upregulating ACSL1 (Hong et al., 2024). Similarly, knocking out augmenter of liver regeneration (ALR) promotes macrophage activation and ferroptosis by altering carnitine palmitoyltransferase 1A (*CPT1A*)-dependent lipid metabolism (Zhang et al., 2024a). These changes accelerate DKD-related kidney damage, providing the functional evidence for the pathogenic role of ferroptosis (Hong et al., 2024; Zhang et al., 2024a).

At the clinical level, renal biopsy samples from patients with DKD exhibit iron deposition, increased lipid peroxidation, and reduced *GPX4* expression (Kim et al., 2021). Molecular pathological analyses reveal that high reduction of tubular *ACSL4* is associated with a rapid decline in renal function (Shen et al., 2025). Furthermore, alterations in multi-molecular biomarker panels in both serum and urine highly align with ferroptotic signatures, providing strong clinical evidence for its role in DKD pathogenesis and progression (Wu et al., 2023; Wang H. et al., 2024; Zhang et al., 2025; Zhao et al., 2025b).

## 3 Pharmacological profile and anti-ferroptotic mechanisms of SGLT2 inhibitors

Ferroptosis is a regulated form of cell death that can be pharmacologically modulated and has recently been regarded as an important factor in progression of DKD. In the clinical treatment of DKD, SGLT2 inhibitors have demonstrated substantial renal protective effects across many large-scale clinical trials (Martinez Leon et al., 2026). These benefits appear to be at least partially independent of their glucose-lowering actions. Emerging evidence indicates that these effects may be partly due to the regulation of ferroptosis-related pathways. Experimental studies and multi-omics analyses show that SGLT2 inhibitors influence the ferroptosis network at several levels, including metabolic reprogramming, maintenance of iron homeostasis, and strengthening of antioxidant defenses. To provide a comprehensive framework, this section first outlines the systemic pharmacological basis of SGLT2 inhibitors. It then systematically summarizes the multilevel mechanisms by which these drugs regulate ferroptosis.

### 3.1 System pharmacology of SGLT2 inhibitors

The first SGLT2 inhibitors came from phlorizin, a natural plant compound. But it broke down too fast in the human body (Tian et al., 2021). To fix this, scientists used a stable C-glucoside bond. This design led to the modern SGLT2 inhibitors we use today, including dapagliflozin, empagliflozin, canagliflozin, and ertugliflozin. This new structure makes the drugs very stable. 1t also makes them highly selective for SGLT2 rather than SGLT1, which avoids bad side effects in the gut (Meng et al., 2008). These drugs mainly work in the proximal tubule. They stop the kidney from taking back sodium and glucose. Because of this, patients lose about 60–80 g of glucose in their urine every day. This also causes a mild loss of water (Wright et al., 2011; Ferrannini et al., 2014). Patients can simply take these pills once a day because they stay active in the blood for 10–13 h. The liver breaks them down, so they are quite safe even for people with moderate kidney disease (Scheen, 2015; Fioretto et al., 2018). They do have some side effects. Genital fungal infections are the most common ones. A rare but serious risk is euglycemic diabetic ketoacidosis (euDKA), especially when patients lack insulin (Perry et al., 2019; Scheen, 2020). People used to worry that these drugs might cause bone fractures, but recent studies show this is not a real danger (Blau et al., 2018; Cowan et al., 2022). Inside the kidney, blocking SGLT2 pushes more sodium to the macula densa. This helps restore the normal tubuloglomerular feedback (TGF). As a result, the incoming blood vessels narrow down. This drops the high pressure inside the glomerulus, which directly reduces proteinuria (Heerspink et al., 2020). Also, since the tubule cells do less work, they need less adenosine triphosphate (ATP) and oxygen. This relieves local lack of oxygen (Vallon and Thomson, 2017). These drugs also help the kidneys clear out uric acid. This is important because it lowers oxidative stress in the whole body (Scheen, 2020; Akbari et al., 2022).

SGLT2 inhibitors do more than just fix kidney blood flow. They actually change how the whole body uses energy. This gives strong protection to multiple organs. The water loss caused by the drugs mainly comes from tissue fluids. It does not drop the blood volume too fast, so the sympathetic nervous system stays quiet. The drugs also lower blood pressure and make blood vessels more flexible. Together, these changes greatly cut down heart failure hospitalizations (Zinman et al., 2015; Cinti et al., 2025). Losing sugar in urine every day means losing calories. This creates an energy shortage. To survive, the body starts to burn stored fat, much like what happens when a person is fasting (Omori et al., 2019; Osataphan et al., 2019). Instead of burning glucose, cells turn to fatty acids and ketones, such as β-hydroxybutyrate (β-HB). This fuel switch is very helpful. It keeps the energy output of mitochondria steady and promotes autophagy to clear cell waste (Mima, 2022; Guo C. et al., 2025). In the liver, these drugs turn down the genes for making new fat, such as acetyl-CoA carboxylase (*ACC*), fatty acid synthase (*FAS*), and sterol regulatory element-binding protein 1c (*SREBP1c*). Because of this, the liver stores less fat. Both animal tests and patient data show that SGLT2 inhibitors can clearly improve fatty liver diseases (Komiya et al., 2016; Kim et al., 2019). Scientists are still discussing if these are direct drug effects or just results of better overall health. But one thing is clear. Fixing the energy balance of the whole body is a key reason why these drugs are so successful in treating heart and kidney diseases (Cinti et al., 2025).

### 3.2 Multilevel regulation of ferroptosis by SGLT2 inhibitors

Representative SGLT2 inhibitors include empagliflozin, dapagliflozin, and canagliflozin. Clinical studies have consistently shown that these drugs reduce proteinuria and slow the decline of estimated glomerular filtration rate (eGFR) (Martinez Leon et al., 2026). Importantly, their renoprotective effects are at least partly independent of glycemic control. With the deepening understanding of the role of ferroptosis in the pathogenesis of DKD, increasing experimental and omics-based evidence suggests that the role of SGLT2 inhibitors extends beyond metabolic and hemodynamic regulation (Martínez-Rojas and Bobadilla, 2025; Tsai et al., 2025). These drugs appear to intervene in the ferroptosis cascade at multiple stages. Such effects include suppression of upstream activation signals, reduction of metabolic substrates in intermediate steps, and restoration of downstream antioxidant defense systems.

#### 3.2.1 Upstream: hypoxia response and iron transport

Chronic hypoxia and disordered iron metabolism in the renal microenvironment of diabetes reinforce each other and act as key triggers of ferroptosis. SGLT2 inhibitors reduce ferroptosis susceptibility at an early stage by improving the balance between local oxygen supply and demand and by directly targeting iron transport.

SGLT2 inhibitors correct the abnormal activation of hypoxia-inducible factor-1α (HIF-1α) (Wang YH. et al., 2024). As a central transcription factor to mediate cellular adaptation to hypoxia, early upregulation of HIF-1α may play a compensatory protective role (Jiang et al., 2020). With persistent hypoxia, HIF-1α remains activated and drives abnormal expression of downstream genes, including heme oxygenase-1 (*H O -1*) (Zhai et al., 2023). Under physiological conditions, HO-1 has anti-inflammatory effects (O'Rourke et al., 2024). However, in DKD, its pathological overexpression accelerates heme breakdown and releases large amounts of labile iron. This iron promotes Fenton reactions and increases lipid peroxidation (Feng et al., 2021). In preclinical murine models and cultured human renal tubular cells, dapagliflozin has been shown to significantly suppress excessive activation of the HIF-1α/HO-1 axis, thereby reducing endogenous iron release and limiting the substrate supply for Fenton reaction (Wang YH. et al., 2024).

SGLT2 inhibitors also promote iron efflux through mechanisms that do not rely on transcription. Solute carrier family 40 member 1 (*SLC40A1)*, also called ferroportin 1,is the only known membrane protein that exports intracellular iron into the circulation (Montalbetti et al., 2013). Its stability directly affects cellular iron levels. In high sugar environments,*SLC40A1* protein gets degraded by ubiquitin, which leads to extra iron inside the cells (Zhang et al., 2024b). In diabetic mice and HK-2 cell models, dapagliflozin binds to *SLC40A1* and stabilizes it. This blocks the degradation process and restores iron export (Huang et al., 2022). Consequently, iron levels drop inside the cell, and *GPX4* expression returns (Huang et al., 2022; Zhang et al., 2024b). Experiments show that silencing *SLC40A1* weakens the drug’s effect (Huang et al., 2022). This confirms that stabilizing *SLC40A1* is key to the treatment.

#### 3.2.2 Midstream: lipid metabolism and energy reprogramming

Ferroptosis is closely linked to PUFA peroxidation and mitochondrial oxidative stress. The cellular lipid load mainly depends on the balance between *de novo* lipogenesis (DNL) and fatty acid β-oxidation (FAO). Mitochondria use FAO to break down fatty acids, which limits peroxidation. When FAO fails, leftover lipids (especially PUFAs) gather in the cells. This reduces ATP production and increases exposure to ROS, making kidney cells sensitive to ferroptosis (Chen et al., 2021). SGLT2 inhibitors help by boosting FAO and mitochondrial energy. Through these effects, they cut down the accumulation of PUFAs and ROS to slow ferroptotic progression (Vallon and Thomson, 2017). In animal models of DKD, drugs like canagliflozin, dapagliflozin, and empagliflozin increase FAO enzymes, improve how kidneys handle lipids and reduce injury (Cai et al., 2020; Lu et al., 2022; Yang et al., 2024). These results show that restoring FAO capacity is important. *CPT1A* is a rate-limiting enzyme for FAO and plays a central role in this process (Miguel et al., 2021). *In vivo* studies using diabetic mice and *in vitro* experiments demonstrate that canagliflozin boosts the expression of *CPT1A*, making FAO more efficient and relieving stress (Gan et al., 2024). The transcription factor forkhead box protein A1(*FOXA1*) also regulates FAO (Xie et al., 2024). The overexpression of *FOXA1* increases *CPT1A* levels and weakens ferroptosis, while *FOXA1* silencing has the opposite effect (Gan et al., 2024). Besides impaired FAO, continuous activation of DNL may also increase PUFA synthesis. This provides substrates for membrane phospholipid peroxidation and makes cells more sensitive to ferroptosis. Malonyl-CoA produced by *ACC* can also inhibit *CPT1A* activity. This increases lipid synthesis while weakening the ability to clear fatty acids (Choi et al., 2007). Although SGLT2 inhibitors are proven to block the *SREBP1c*-*ACC* axis in metabolic tissues of animal models, we still need further proof to know if they directly affect tubular ferroptosis by regulating DNL in DKD (Kim et al., 2019).

β-HB is made during nutrient deficiency. It supports ATP production through mitochondrial oxidative phosphorylation and keeps cellular energy steady (Veneti et al., 2023). Calcium/calmodulin-dependent protein kinase kinase 2 (CaMKK2) is an important metabolic regulator involved in mitochondrial function and fatty acid metabolism (McAloon et al., 2024). In preclinical models, an increase in renal β-HB induced by dapagliflozin acts as a key signaling event (Tian et al., 2025). This change suppresses abnormal CaMKK2 activation, improves mitochondrial calcium balance, lowers ROS production, and blocks activation of the lipid peroxidation cascade (Tian et al., 2024; Tian et al., 2025).

#### 3.2.3 Downstream: antioxidant and antifibrotic pathways

In the downstream of the ferroptosis cascade, SGLT2 inhibitors establish a defense system with both antioxidant and anti-fibrosis functions by rebuilding the key transcription regulation network. These drugs primarily restore cellular redox homeostasis by activating the AMP-activated protein kinase (AMPK)/*NRF2* signaling axis. In both diabetic mice and high-glucose stimulated HK-2 cells, empagliflozin has been shown to enhance phosphorylation of AMPK at Thr172, which in turn promotes nuclear translocation of *NRF2* (Lu et al., 2023). The activation of *NRF2* turns on several classical anti-ferroptotic genes, including *GPX4* and ferritin heavy chain 1 (*FTH1*). Importantly, it also upregulates glutamate–cysteine ligase modifier subunit (*GCLM*) (Tsai et al., 2025). In DKD, the consumption of *GCLM* limits GSH synthesis and leads to functional inactivation of *GPX4*(Lu, 2013)*.* Based on *in vitro* and *in vivo* DKD models, by restoring *GCLM* expression, SGLT2 inhibitors ensure the availability of substrates for GSH synthesis at its source (Lu, 2013; Tsai et al., 2025). This reactivates the *GCLM*–GSH–*GPX4* axis, promotes efficient detoxification of lipid peroxides, and preserves mitochondrial integrity (Tsai et al., 2025).

Beyond antioxidant reinforcement, SGLT2 inhibitors also prevent the transition from ferroptotic injury to fibrotic remodeling by suppressing solute carrier family 7 member 7 (*SLC7A7*). *SLC7A7* is a cationic amino acid transporter which is abnormally upregulated in the kidney tissue of DKD and shows a positive correlation with the ferroptosis driver *ACSL4* and fibrosis markers, including α-smooth muscle actin (α-SMA) and collagen type Ia 1 (*Col1a1*) (Zhao et al., 2025a). The overexpression of *SLC7A7* exacerbates metabolic imbalance, enhances *ACSL4*-dependent membrane lipid remodeling, and promotes epithelial–mesenchymal transition (EMT) (Doll et al., 2017; Zhao et al., 2025a). In diabetic rat models and cultured renal proximal tubular cells, empagliflozin has been shown to selectively downregulate the expression of *SLC7A7*, thereby destroying the *SLC7A7*-*ACSL4* metabolic axis and significantly reducing renal interstitial fibrosis (Zhao et al., 2025a). Through these transcriptional effects, redox homeostasis can be maintained even under persistent upstream stress.

Overall, SGLT2 inhibitors play a precise multi-level pharmacological regulatory role in the ferroptosis pathway (Table 1). At the start, they restore iron efflux and reduce endogenous iron accumulation. Midstream, they increase fatty acid β-oxidation and stop the cell from making oxidized lipids. At the downstream level, they rebuild antioxidant and antifibrotic defense networks to improve cellular resistance to oxidative stress. Together, these coordinated actions provide a mechanistic explanation for the renal protective effects of SGLT2 inhibitors in DKD and offer a strong biological basis for their clinical application in the treatment of ferroptosis-related kidney injury.

**TABLE 1:** Integrated layered mechanisms by which SGLT2 inhibitors regulate ferroptosis in diabetic kidney disease.

Regulatory level	Core mechanism	Key molecular targets	Specific biological effects	Representative agents and references
Upstream: blockade of initiating triggers	Suppression of hypoxia-driven iron release	HIF-1α/HO-1↓	Attenuates pathological overactivation of HIF-1α, downregulates HO-1–mediated heme degradation, and limits endogenous iron release	Dapagliflozin (Wang et al., 2024b)
	Preservation of iron efflux capacity	*SLC40A1*(FPN1)↑	Directly binds to and stabilizes *SLC40A1*, prevents ubiquitin-mediated degradation, promotes cellular iron export, and reduces intracellular iron burden	Dapagliflozin (Huang et al., 2022)
Midstream: metabolic reprogramming	Ketone body–mediated antioxidative modulation	β-HB↑/CaMKK2↓	Enhances β-hydroxybutyrate production, suppresses CaMKK2 signaling, improves mitochondrial calcium homeostasis, and reduces ROS generation	Dapagliflozin (Tian et al., 2025)
	Enhancement of fatty acid oxidation	*FOXA1*/*CPT1A*↑	Activates the transcription factor *FOXA1*, upregulates *CPT1A*, enhances mitochondrial FAO,accelerates lipid consumption, and reduces lipid accumulation	Canagliflozin (Gan et al., 2024)
Downstream: reinforcement of cellular defense	Activation of transcriptional antioxidant networks	AMPK/*NRF2*↑ *GCLM*↑	Activates AMPK phosphorylation and promotes *NRF2* nuclear translocation; upregulates *GCLM* to support *de novo* GSH synthesis; increases *GPX4* and *FTH1* expression to enhance lipid peroxide detoxification and iron sequestration	Empagliflozin (Lu et al., 2023; Tsai et al., 2025)
	Inhibition of fibrotic remodeling	*SLC7A7*/*ACSL4*↓	Downregulates the amino acid transporter *SLC7A7*, indirectly suppresses *ACSL4*-mediated membrane lipid remodeling, and blocks fibroblast-like transdifferentiation	Empagliflozin (Zhao et al., 2025a)

## 4 Clinical biomarkers and translational perspectives

Although accumulating cellular and animal studies support the involvement of ferroptosis in the progression of DKD,clinical investigations remain at an early stage. The current evidence is mainly derived from analyses of circulating and urinary biomarkers, as well as small-scale observational studies. These studies show that the metabolic characteristics associated with ferroptosis are closely related to the occurrence and progression of DKD. If a clinically applicable ferroptosis biomarker analysis framework can be established, it is expected to quantitatively evaluate ferroptosis activity and may help achieve more accurate disease stratification and therapeutic effect prediction.

### 4.1 Serum iron metabolism indicators

In the current clinical laboratory system, several routine parameters can indirectly reflect ferroptosis-related risk. Elevated serum ferritin levels have been reported to be independently associated with the development and progression of chronic kidney disease (CKD) in patients with type 2 diabetes. This finding indicates that excessive iron storage may contribute to CKD pathogenesis (Wu et al., 2020). A phenotype defined by low serum iron together with high serum ferritin is also independently associated with a higher risk of DKD. This pattern highlights the disrupted iron distribution and utilization in DKD (Huang et al., 2025).

Large studies agree with this. A study of National Health and Nutrition Examination Survey (NHANES) found that serum iron, total iron-binding capacity, and transferrin saturation were lower in people with DKD,albuminuria, and reduced eGFR. But ferritin had a J-shaped link to DKD (Gong et al., 2025). This means the problem is iron imbalance, not just iron overload.

Still, the clinical utility of serum iron indicators remains limited. Ferritin changes because of inflammation, not just kidney issues (Wu et al., 2020; Huang et al., 2025). It fails to show ferroptosis at the tissue level. Because of this, these indicators are mainly suitable for risk assessment at the population level, but not for measuring ferroptosis in the kidney.

### 4.2 Urinary and renal-specific biomarkers

To fix the problem that existing biomarkers are not specific enough, recent studies focus on urine indicators related to kidney ferroptosis. Data shows that urinary ferritin is closely linked to tubular injury, and it often rises earlier than the appearance of proteinuria. These findings suggest that urinary ferritin not only reflects iron overload in the renal tubule, but also may be used as a sensitive indicator of early renal tubular injury in DKD. So, urinary ferritin is expected to make up for the slow response of the urinary albumin-to-creatinine ratio (UACR) (Huang et al., 2025).

Besides single markers, combining them is effective. A prospective observational study reported that a biomarker panel consisting of *ACSL4*, *GPX4*, MDA and ROS has good accuracy. The area under the curve (AUC) was 0.804. Notably, in patients with obvious proteinuria, the levels of *ACSL4*, MDA, and ROS were significantly increased, while the levels of *GPX4* were markedly reduced (Wu et al., 2023). These patterns are highly consistent with the typical ferroptosis characteristics. Furthermore, ferroptosis execution-related proteins such as arachidonate 15-lipoxygenase (*ALOX15*) and *HMOX1* were significantly upregulated in the serum of DKD patients and closely related to inflammatory markers (Zhao et al., 2025b). This finding supports the presence of functional crosstalk between ferroptosis and inflammation.

Epigenetic regulatory factors have also become potential biomarkers. The high expression of histone methyltransferase enhancer of zeste homolog 2 (*EZH2)* is closely related to the progression of DKD. Mechanism studies show that *EZH2* promotes ferroptosis by suppressing *SLC7A11*, highlighting its diagnostic potential (Wang H. et al., 2024). In summary, these findings show that ferroptosis-related signals can be captured at the clinical level and may provide diagnostic, prognostic and stratified value.

### 4.3 Evaluation of current clinical evidence

Based on current research, three consistent clinical characteristics can be identified: (1) systemic iron metabolism indicators suggest that iron homeostatic imbalance (characterized by iron overload or utilization disorders) is positively correlated with the risk of DKD (Wu et al., 2020; Gong et al., 2025; Huang et al., 2025); (2) urinary biomarkers are consistent with the execution process of renal tubular injury and ferroptosis (Huang et al., 2025); (3) changes in multi-molecular biomarker panels are consistent with the severity of proteinuria, reflecting the specific characteristics of the disease stage (Wu et al., 2023; Zhao et al., 2025b). However, there are still some limitations. Most of the existing studies are cross-sectional studies, and there is a lack of longitudinal tracking of ferroptosis biomarkers in the progression of the disease (Wu et al., 2023; Zhang et al., 2025; Zhao et al., 2025b). In addition, biomarker detection lacks standardized methods,and clinically validated thresholds have not been established. Moreover, there is currently no tissue-level gold standard to confirm the renal origin or causal relevance of these biomarkers. Finally, sample sizes are generally limited and there is a lack of multicentre verification. As a result, the current clinical evidence is still mainly descriptive and correlational, rather than predictive or causal.

Although multiple ferroptosis-related genes have been identified in DKD, it should be noted that most clinical biomarkers reflect pathway activity rather than direct ferroptotic execution events.

### 4.4 Precision medicine and patient stratification

DKD shows marked biological heterogeneity, with distinct pathological drivers including metabolic dysregulation, inflammatory amplification, and renal tubular injury (Kim et al., 2021; Li et al., 2023). The core role of ferroptosis in tubular oxidative stress and inflammation amplification suggests the presence of the “ferroptosis-dominated DKD” subtype. Its potential molecular features include elevated urinary ferritin, increased *ACSL4* expression, reduced *GPX4* levels, increased MDA and ROS, and activation of *ALOX15* and *HMOX1* (Wu et al., 2023; Huang et al., 2025; Zhao et al., 2025b).

If confirmed, this subtype would allow ferroptosis biomarkers to predict disease risk and to guide treatment stratification. SGLT2 inhibitors regulate ferroptosis by improving lipid metabolism, maintaining iron balance, and strengthening antioxidant capacity (Yang et al., 2024; Martínez-Rojas and Bobadilla, 2025; Tsai et al., 2025). Based on this mechanistic framework, a testable clinical hypothesis can be proposed. Patients with DKD who show clearly elevated urinary ferritin levels or increased exosomal *ACSL4* expression may respond more strongly to the anti-ferroptotic effects of SGLT2 inhibitors and may gain greater renal benefit. This hypothesis links biomarker screening, subtype definition, and treatment response evaluation, and it supports clinical translation.

In summary, ferroptosis-related biomarker systems provide useful information for DKD clinical research and support a shift from simple risk assessment to disease stratification and precision therapy. However, it is necessary to carry out large, multicentre, longitudinal studies to build a standardized testing platform and define clinically usable thresholds for stable clinical application.

## 5 Discussion

Multiple lines of evidence from cellular, animal, and clinical studies show that ferroptosis is involved in the occurrence and progression of DKD. Ferroptosis damages renal tubular epithelial cells by causing iron overload and excessive lipid peroxidation (Jiang et al., 2021). These processes are closely related to the structural remodeling and progressive decline of kidney function (Kim et al., 2021). Within the multi-factorial pathological landscape of DKD, ferroptosis is a critical biological link connecting metabolic disorders, oxidative stress, and tissue fibrosis (Li et al., 2023; Lyu et al., 2025). This linkage provides a mechanistic basis for renal tubular vulnerability and sustained progression of the disease. The renoprotective effects of SGLT2 inhibitors have been fully verified in large-scale clinical trials and are partially independent of glucose lowering (Mazzieri and Marcon, 2025; Martinez Leon et al., 2026). As mentioned above, these drugs suppress ferroptosis through a coordinated network. At the upstream level, they correct maladaptive hypoxic responses and iron transport dysfunction. At the midstream level, they reprogram lipid and energy metabolism. At the downstream level, they reinforce transcriptional antioxidant defense networks.

Besides directly controlling iron and lipids, SGLT2 inhibitors also change the body’s overall environment to fight ferroptosis (Mazzieri and Marcon, 2025). For example, they help the kidneys excrete uric acid to lower its blood level (Akbari et al., 2022). This removes a main source of oxidative stress inside the cell, which usually makes lipid peroxidation worse (Verzola et al., 2014). Furthermore, SGLT2 inhibitors reduce inflammation. In DKD, damaged tubule cells activate macrophages and the NOD-like receptor family pyrin domain containing 3 (NLRP3) inflammasome, making a lot of ROS(Fang et al., 2025). By raising β-HB levels and activating AMPK, these drugs stop the inflammasome from forming and change how immune cells use energy. This breaks the vicious circle of inflammation and oxidation (Kim et al., 2020).

At the subcellular level, maintaining mitochondrial health is another key factor in preventing ferroptosis. In DKD, because cells cannot burn fat well, the electron transport chain leaks ROS. At the same time, unused PUFAs build up in cell membranes (Hughes et al., 2025). SGLT2 inhibitors fix this problem by activating the peroxisome proliferator-activated receptor gamma coactivator 1α (*PGC-1α*) pathway to make new and healthy mitochondria (Afsar et al., 2021). Animal studies confirm that this restores mitochondrial function, which directly reduces ROS leakage and stops lipid peroxidation (Guo C. et al., 2025).

Importantly, ferroptosis does not happen alone. It interacts with apoptosis and pyroptosis to form a united death network called the PANoptosome (Lv et al., 2025). In the DKD environment, high blood sugar and lipotoxicity can trigger apoptosis, pyroptosis, and ferroptosis all at once (Guo Y. et al., 2025). These pathways also feed into each other. For example, the large amount of ROS and lipid peroxides from ferroptosis can easily activate inflammasomes to push pyroptosis forward (Wang J. et al., 2025). In the end, these different cell death types join together to form a united death network called the PANoptosome (Lv et al., 2025). Because these pathways are connected so tightly, an important question remains: does blocking ferroptosis simply cause other cell death pathways to activate and take over?

Although many cell and animal studies support these mechanisms, we still lack long-term clinical data to track ferroptosis during DKD progression. Existing biomarkers mainly reflect systemic iron metabolism or oxidative stress, and have limited information on kidney-specific ferroptotic events (Wu et al., 2023). Emerging approaches, including liquid biopsy, urinary exosome proteomics, and single-cell mapping, offer promising tools for ferroptosis detection. However, standardized analytical platforms, diagnostic thresholds, and population-specific criteria have yet to be established (Mu et al., 2024; Zhou et al., 2025). Instead of starting with genetically changed mice, future research must adopt a “human-first” approach (Podesta and Steiger, 2026). By analyzing patient-derived tissues and fluids with advanced multiomic tools, researchers can identify the key ferroptosis-related pathways or targets and molecular subtypes in human DKD. Once these real-world targets are clear, selectively chosen animal models or human kidney organoids can be used to validate how SGLT2 inhibitors work on these specific pathways. Ultimately, this reverse translational process will help design precision treatments for different patient groups, making sure the anti-ferroptosis strategies actually benefit real patients in clinical settings. Finally, strict clinical studies are necessary to find the best time window to start anti-ferroptosis treatment.

## 6 Conclusion

Experimental evidence suggests that SGLT2 inhibitors may inhibit ferroptosis through the regulation of iron balance, lipid metabolism, and antioxidant systems. These findings provide new insights into the kidney-protective effects of SGLT2 inhibition. At present, however, the clinical significance of targeting ferroptosis has not been fully confirmed.

More translational and clinical studies are needed to determine whether ferroptosis can be used as a treatable pathway in DKD and to define its role in the long-term benefits of SGLT2 inhibitors. A clearer understanding of ferroptosis-related pathways may help improve disease stratification and support the development of targeted strategies for renal protection.

## Glossary

4-HNE: 4-Hydroxynonenal
AA-PE: Arachidonic acid-phosphatidylethanolamine
*ACC*: Acetyl-CoA carboxylase
*ACSL1*: Acyl-CoA synthetase long-chain family member 1
*ACSL4*: Acyl-CoA synthetase long-chain family member 4
AdA-PE: Adrenic acid-phosphatidylethanolamine
AHA: American Heart Association
AKI: Acute kidney injury
*ALOX15*: Arachidonate 15-lipoxygenase
ALR: Augmenter of liver regeneration
AMPK: AMP-activated protein kinase
*ATF4*: Activating transcription factor 4
ATP: Adenosine triphosphate
AUC: Area under the curve
β-HB: β-Hydroxybutyrate
CaMKK2: Ca^2+^/calmodulin-dependent protein kinase kinase 2
CHOP: C/EBP homologous protein
CKD: Chronic kidney disease
*Col1a1*: Collagen type I alpha 1
*CPT1A*: Carnitine palmitoyltransferase 1A
DAMPs: Damage-associated molecular patterns
DHODH: Dihydroorotate dehydrogenase
DKD: Diabetic kidney disease
DMT1: Divalent metal transporter 1
DNL: *De novo* lipogenesis
eGFR: Estimated glomerular filtration rate
eIF2α: Eukaryotic initiation factor 2 alpha
EMT: Epithelial-mesenchymal transition
ESKD: End-stage kidney disease
euDKA: Euglycemic diabetic ketoacidosis
EZH2: Enhancer of zeste homolog 2
FAO: Fatty acid β-oxidation
*FA*S: Fatty acid synthase
*FOXA1*: Forkhead box A1
FPN1: Ferroportin 1
FSP1: Ferroptosis suppressor protein 1
*FTH1*: Ferritin heavy chain 1
GBD: Global Burden of Disease
*GCLM*: Glutamate-cysteine ligase modifier subunit
*GPX4*: Glutathione peroxidase 4
GSH: Glutathione
HIF-1α: Hypoxia-inducible factor 1 alpha
HK-2: Human kidney-2
*HMOX1*: Heme oxygenase 1 (protein: HO-1)
Hrd1: HMG-CoA reductase degradation protein 1
IDF: International Diabetes Federation
IL-6: Interleukin 6
*LPCAT3*: Lysophosphatidylcholine acyltransferase 3
MDA: Malondialdehyde
*NCOA4*: Nuclear receptor coactivator 4
NHANES: National Health and Nutrition Examination Survey
*NLRP3*: NOD-like receptor family pyrin domain containing 3
NOD: Non-obese diabetic
*NRF2*: Nuclear factor erythroid 2–related factor 2
PANoptosis: Pyroptosis apoptosis and necroptosis
PERK: Protein kinase RNA-like endoplasmic reticulum kinase
*PGC-1α*: Peroxisome proliferator-activated receptor gamma coactivator 1-alpha
PLOOH: Phospholipid hydroperoxides
PRMT6: Protein arginine methyltransferase 6
PUFA: Polyunsaturated fatty acids
PUFA-PE: Polyunsaturated fatty acid-phosphatidylethanolamine
RAS: Renin–angiotensin system
ROS: Reactive oxygen species
SGLT1: Sodium-glucose cotransporter 1
SGLT2i: Sodium–glucose cotransporter 2 inhibitors
*SLC3A2*: Solute carrier family 3 member 2
*SLC7A7*: Solute carrier family 7 member 7
*SLC7A11*: Solute carrier family 7 member 11
*SLC40A1*: Solute carrier family 40 member 1
*SREBP1c*: Sterol regulatory element-binding protein 1c
STZ: Streptozotocin
T2DM: Type 2 diabetes mellitus
TFR1: Transferrin receptor 1
TGF: Tubuloglomerular feedback
UACR: Urinary albumin-to-creatinine ratio
XBP1: X-box binding protein 1
xCT: Core subunit of System Xc-
ZIP14: ZRT/IRT-like protein 14
α-SMA: Alpha-smooth muscle actin.
